# Pre-natal nutrition education: Health care providers’ knowledge and quality of services in primary health care centres in Lagos, Nigeria

**DOI:** 10.1371/journal.pone.0259237

**Published:** 2021-11-09

**Authors:** Hanifat Abisola Ibikunle, Ifeoma Peace Okafor, Adebola Afolake Adejimi

**Affiliations:** Department of Community Health & Primary Care, College of Medicine, University of Lagos, Lagos, Nigeria; Waikato Institute of Technology, NEW ZEALAND

## Abstract

**Introduction:**

A healthy and balanced diet is very important during pregnancy. By enhancing maternal nutritional status, healthcare providers can lower the risks of pregnancy complications and adverse birth outcomes.

**Objectives:**

To assess the pre-natal nutrition knowledge and services rendered by healthcare providers in antenatal clinics at Primary Health care Centres in Lagos, Nigeria.

**Methodology:**

This was a cross-sectional study carried out in June—September 2019. Self-administered questionnaires were used to collect data on nutritional knowledge from 391 nurses and midwives who provided pre-natal nutrition education. Direct observation with checklist was used to assess the nutrition education services at the ANC clinics and covered: adequacy of the venue, availability/use of nutrition education resources, education content and teaching strategies. EPi-Info version 3.5 was used for data analysis. Inferential statistics such as chi square and multiple logistic regression were used to determine associated factors and predictors of nutrition knowledge. The level of significance was set at 5% (p<0.05).

**Results:**

The mean age of respondents was 41.71±10.33years, all were females; 44.8% were Registered Nurses, 12.0% were Registered Midwives, and 23.8% had a B.Sc. in Nursing. Majority (81.3%) had taken a nutrition course in nursing training program, 62.1% as elective classes. Majority (81.1%) had good knowledge of prenatal nutrition. Respondents who were older (51-60years) (p<0.001), single (p<0.001) and Christian (p = 0.001) had significantly better knowledge. Nurses who had University degrees (p<0.001), of higher cadre (p<0.001), more years of practice (p<0.001) and involved in treating severely malnourished children (p = 0.013) were also significantly more knowledgeable. Respondents below 40 years (OR 0.104, CI 0.049–0.218) and those with 10 years or less of practice (OR 0.189, CI 0.092–0.387) had less odds of having good nutrition knowledge. Being single (OR 8.791, CI 3.125–24.731), and Christian (OR = 5.810, CI: 3.321–10.164) predict good nutrition knowledge. In 39% of the 41 PHCs observed, quality of nutrition education services was inadequate. Gaps were mainly in availability of certain nutrition education facilities/resources and teaching strategies.

**Conclusion:**

The majority of the respondents had good knowledge of pre-natal nutrition. Overall, however, nutrition education services provided by two-fifths of the facilities were inadequate. Interventions to improve prenatal nutrition knowledge of nurses/midwives should be focused more on those who are younger and have less work experience. All necessary equipment required for health service providers to execute their roles as nutrition counselors and educators should also be provided by the government.

## Introduction

Regular antenatal care plays an important role in the identification and reduction of risks to mother and child during pregnancy and is associated with positive child health outcomes [1]. It offers the opportunity for health professionals to discuss and support health behaviours recommended to ensure a healthy pregnancy such as increasing intakes of vegetables, fruits and other food group needs, and abstaining from alcohol and smoking. Health professionals should also increase awareness of important dietary considerations during pregnancy. Pregnant women have increased nutrient requirements [2] and they are more susceptible to food-borne illness due to hormonal changes which may lead to rejection of the foetus [3]. For this, certain advisories are issued against the ‘over-consumption’ of otherwise healthy food and the avoidance of some, totally [4]. Making appropriate food choices during pregnancy, however, can be complicated as pregnant women are the recipients of multiple sources of information and recommendations [5], and consequently may unnecessarily increase levels of anxiety about food choices [6].

A healthy and balanced diet is very important in life time and during pregnancy in particular. The maternal diet must provide sufficient energy and nutrients to meet the mother’s usual requirements, as well as the needs of the growing fetus. A healthy maternal diet enables the mother to maintain her own stores of nutrients required for fetal and infant health as well as for future breastfeeding practices. The main recommendation is to follow a healthy, balanced diet [1]. Pregnancy is a period when women become more aware of the importance of healthy nutrition and seek more nutrition-related information. Compared to preconception period, pregnant women are more eager to know what they should eat and what they should not eat [7]. Preconception nutrition-specific interventions, in particular increased folic acid and multivitamins supplements among pregnant women resulted in positive pregnancy outcomes [8].

By enhancing maternal nutritional status, midwives can help women lower the risks of pregnancy complications and adverse birth outcomes as well as improve maternal health during pregnancy and in the long run [9]. A widely used strategy to improve nutritional status of women during pregnancy is nutrition education and counselling (NEC). Nutrition education and counselling strategies typically focus on enhancing maternal diet quality by increasing the diversity and amount of foods consumed, adequate weight gain through consumption of sufficient and balanced protein and energy, and the consistent and continued use of micronutrient supplements, food supplements or fortified foods [1]. Prenatal NEC interventions in pregnant adolescents positively impact nutrition knowledge and diet quality [10]. Interventions also modestly improve gestational weight gain and birthweight. Another review of studies, largely in high‐income countries reported variations in effectiveness of different intervention methods on gestational weight gain. Authors concluded that the studies were few and the evidence was of insufficient quality to draw specific conclusions strong enough to make evidence-based recommendations in clinical antenatal care [11]. A later review shows that NEC significantly reduce the risk of anaemia in late pregnancy, increase birthweight and lower the risk of preterm delivery, but the effect on risk of low birthweight is not significant. These effects are greater when given with nutrition support such as food or micronutrient supplementation or nutrition safety nets [12].

The provision of nutrition advice by health professionals to pregnant women is limited, with lack of practitioner education being indicated as one of the obstacles to the provision of such advice [10, 11]. Research has reported that nutrition education received by doctors is inadequate [13–15], however, midwives’ nutrition education and services has received little attention. Midwives constitute a major primary care provider of prenatal care. They have been reported as the most frequent source of health information for pregnant women [16] and their role in the provision of nutrition advice is clearly defined in the International Confederation of Midwives Essential Competencies [17]. However, they lack adequate nutrition knowledge and skills [18].

A study investigating nutrition education within midwifery programmes using a mixed-methods approach incorporating online surveys and interviews with the course coordinators of the programmes identified some gaps [19]. Although all surveyed programmes included nutrition content within their curricula, topics taught varied, and the number of total hours was low. The education seemed to be medically oriented and lacked focus on developing nutrition assessment skills or providing practical nutrition training [19].

Therefore, nutrition education is an essential consideration to optimize health of women of reproductive age in addition to pregnancy outcomes. Nutrition education programmes are important as they are targeted at enhancing subjects’ dietary intakes by promoting behavioral changes such as food choice and cooking ability, goal-setting, motivation, and supporting the efforts for a change [20]. There is dearth of published data on nutrition education among health workers in Nigeria and indeed the African Region. The objective of this study therefore was to assess the nutritional knowledge and the services rendered by healthcare providers (nurses and midwives) in antenatal clinics at Primary Health care Centres (first level of care) in Lagos State where many expectant women seek antenatal care. Findings will give an overall baseline situation and expose gaps for intervention.

## Materials and methods

### Study design and setting

This study used a cross-sectional design which assessed nutritional knowledge and services rendered by healthcare providers in antenatal clinics at Primary Healthcare Centres (PHCs) in Lagos State, Nigeria. Lagos State is made up of 20 Local Government Areas (LGAs), ie the District level. Sixteen of them are urban while four are rural. There are 288 PHCs, 165 of them are located in the Urban LGAs all of which were included in the study. At each of the PHCs, nutrition education is conducted by the designated health workers during the antenatal clinics. Nutrition education sessions conducted on each clinic day emphasize the importance of each food group to the health of the pregnant women and the baby with demonstration of food types. The study population comprised nurses and midwives in the urban PHCs in Lagos State.

### Sampling method and data collection

At the time of the study, a total of 473 nurses/midwives were working in the 165 urban PHCs. Excluded from the study were trainee nurses/midwives and those who were officially on leave during the period of data collection. Only those involved in nutrition education at the ANC clinics were included. Four hundred and ten (410) nurses from all the 165 PHCs met the inclusion criteria and were given questionnaires. Subsequently, 391 were returned and adequately filled for analysis. Eleven were not returned and eight were inadequately filled for analysis. A quarter (41) of the 165 urban PHCs were observed with aid of observation checklists. They were selected by simple random sampling.

A structured, pre-tested, self-administered questionnaire was used to collect data and observation checklist to assess nutrition education services. The tools were developed from previous studies [9, 21–24]. Pretesting was done among 20 nurses/midwives in selected urban PHCs in neighboring Ogun State. Validation was done by experts in the field (two nursing school tutors) in the School of Nursing in Lagos University Teaching Hospital. Questionnaires were distributed by trained research assistants and retrieved from a central collection point in each PHC. Direct observations were done by the lead researcher and four trained research assistants on ANC clinic days which were usually held twice weekly in the PHCs. Observations commenced in the morning as soon as the clinic starts and covered the following: general assessment of the nutrition education venue such as availability of permanent seats, organization and conduciveness of the learning environment, and topics covered such as food and hand hygiene, balanced diet, specific nutrient needs in pregnancy, nutrition in the presence of other health conditions among others. Other areas of observation include availability of guidelines and nutrition education resources such as visual/audio aids, posters/charts and the teaching strategies used such as food demonstration in addition to active participation of the pregnant women. The observer usually sat with the pregnant women and sought further information from the nurses as needed.

### Data analysis

The EPi-Info version 3.5 was used for data analysis. Descriptive statistics was calculated for all variables. For continuous variables, mean and standard deviation were calculated. The nutrition knowledge level was assessed using a set of questions. Each correct answer was awarded one mark, otherwise zero. Scores were converted to percentages where (<50%) was considered poor knowledge and (≥50%) was considered good nutrition knowledge. This cut-off was guided by the Nigerian nursing and midwifery training scoring system where a minimum score of 50% is considered good. Bivariate analysis was done using Chi-square test and P-value was set at 0.05. The dependent variable was level of nutrition knowledge, while independent variables were socio-demographic and occupational variables. As these independent variables could act as confounders, those which showed statistically significant associations were subsequently entered into a multi-variate regression model to ascertain predictors of good nutrition knowledge.

On the assessment of quality of nutrition education services, all components on the assessment of nutrition education in the observation checklist were added together and analyzed.

The categories of services were established using point scale (1 = Yes, 0 = No). Assessment of nutrition education services was classified into adequate and inadequate level. PHCs providing adequate services were categorized by scoring 38 components in the checklist and scores converted to percentages. PHCs scoring less than 30 out of the 38 components (<80%) were classified as providing inadequate nutritional services while PHCs scoring at least 30 out of the 38 components (≥80%) were classified as providing adequate nutritional services. This is an adaptation of assessment of antenatal care services in the country by previous researchers [24].

Ethical approval (ADM/DCST/HREC/APP/2949) was obtained from the Health Research Ethics Committee of the Lagos University Teaching Hospital. Permission was granted from the Primary Health Care Board and Chairmen and Medical officers in all the LGAs used for the study.

Written informed consent was obtained from all the respondents after explaining the nature of the study and its objectives. Assurance was given that participation was purely voluntary without negative consequences for non-participation. Confidentiality of the participants’ responses were guaranteed by using numbers rather than names to identify the respondents and the questionnaires. The participants were given the assurance that the data collected were purely for research purposes. Also, the benefits of the study to the workers and the importance of providing a feedback to them were clearly explained. For the observations, the health providers’ and clients’ permission were sought and obtained verbally.

## Results

### Socio-demographic characteristics of respondents

The socio-demographic characteristics of respondents are presented in Table 1. The mean age of respondents was 41.71±10.33years, Majority 71.9% were married/cohabiting, Christians (61.9%), and of Yoruba ethnic group (68.3%). About 44.8% of the respondents were registered Nurse/Midwife, 12.0% were registered Midwife, while 23.8% were Bachelor of science in Nursing (BNSc) degree holders. Their professional cadres ranged from Deputy Director of Nursing Services (DDNS), to Nursing Officer II (NOII). The mean years of practice for respondents was 16.74±10.54years. Slightly more than a quarter (26.3%) had been practicing for more than 20 years.

**Table 1:** Socio-demographic characteristics of respondents.

Variable	Frequency (n = 391)	Percentage (%)
**Age(years)**		
21–30	47	12.0
31–40	169	43.2
41–50	62	15.9
51–60	113	28.9
Mean age = 41.71±10.33years		
**Marital Status**		
Married/cohabiting	281	71.9
Not married	123	28.1
**Religion**		
Christianity	242	61.9
Islam	149	38.1
**Ethnicity**		
Yoruba	267	68.3
Igbo	105	26.9
Others	19	4.9
**Qualification**		
RN	30	7.7
Post-basic	31	7.9
Registered nurse/Midwife	175	44.8
Registered midwife	47	12.0
BNSc	93	23.8
MSc and Postgraduate	15	3.8
**Cadre**		
DDNS	30	7.7
ADNS	73	18.7
CNO	40	10.2
PNO	88	22.5
SNO	33	8.4
NOI	57	14.6
NOII	70	17.9
**Duration of Practice (year)**		
1–5	95	24.3
6–10	60	15.3
11–15	82	21.0
16–20	51	13.0
>20	103	26.3
Mean years of practice	16.74±10.54	

DDNS-Deputy director nursing services; ADNS-Assistant director nursing services; CNO-Chief nursing officer; PNO- Principal nursing officer; SNO- Senior nursing officer; NO- Nursing officer I; NOII- Nursing officer II

Table 2 is on the nutritional knowledge of respondents. A majority (81.1%) of the respondents knew that inadequate and unbalanced nutrition during pregnancy affects both mother and baby. Only about two-fifths (38.9%) knew that Iron need increases during pregnancy and fewer proportions knew Calcium and Zinc. Majority (75.4%) indicated that there should be daily increase in the consumption of fruits and vegetables during pregnancy. The major cause of neural tube defective births (inadequate folate/Folic Acid) was known by two-thirds (66.2%) of the health workers.

**Table 2:** Respondents’ knowledge of nutrition in pregnancy.

Variable n = 391	Frequency	Percentage %
**Typical types of food that should make up regular/house diet** *		
Foods low in fat	104	26.5
Foods low in Cholesterol and Sodium	88	22.5
Food high in fiber	199	50.9
I do not know	9	2.3
Others carbohydrate, protein	21	5.5
**Daily portions of milk and dairy products consumed in pregnancy**		
At least 2 portions	242	61.9
At least 3 portions	82	21.0
At least 4 portions	48	12.3
I do not know	19	4.9
**Inadequate and unbalanced nutrition during pregnancy affect:**		
Mother only		
Baby only	45	11.5
Mother and Baby	317	81.1
I do not know	29	7.4
**Iron need of woman during pregnancy**		
Decreases	130	33.2
Increases	152	38.9
Neither increases nor decreases	90	23.0
I do not know	19	4.9
**Seafood to be avoided during pregnancy**		
Shrimp	79	20.2
Snail	3	0.8
Tortoise	130	33.2
I do not know	160	40.9
Others (cat fish, smoked fish, crab, jelly fish etc)	19	4.9
**During pregnancy period, there should be daily increase in the consumption of foods such as:** *		
Vegetable	47	12.0
Fruits	118	30.2
Fruit and Vegetables	295	75.4
I do not know	0	0.0
Others (juice, Vitamin C etc)	19	4.9
**During pregnancy there is need for increase in minerals such as** *		
Calcium	170	43.5
Iron	270	69.1
Zinc	112	28.6
Iodine	97	24.8
**Cause of Osteomalacia during pregnancy**		
Inadequate consumption of minerals such as calcium and phosphorus in pregnancy	318	81.3
Inadequate exposure to sunlight	33	8.4
Don’t know	40	10.2
**Overweight/obese mother in the pre-pregnancy period should**		
Lose weight	95	24.3
Reach normal range of BMI	296	75.7
I do not know	0	0.0
**Cause of neural tube defective births**		
Folate/Folic Acid inadequacy in pregnancy	259	66.2
Vitamin B inadequacy	113	28.9
Lack of multivitamin with 400 micrograms	19	4.9

*Multiple response

Results indicating the association between respondent’s socio-demographic characteristics and their nutritional knowledge is presented in Table 3. There was a statistically significant association between socio-demographic characteristics namely age, marital status, religion and ethnicity of healthcare providers and level of nutrition knowledge.

**Table 3:** Socio-demographic factors associated with nutrition knowledge.

Variable	Nutrition knowledge		X^2^	p-value
	Poor (n = 74)	Good (n = 317)		
**Age(years)**				
21–30	7(14.9)	40(85.1)	53.762	<0.001^#^
31–40	58(34.3)	111(65.7)		
41–50	9(14.5)	53(85.5)		
51–60	0(0.0)	113(100.0)		
**Marital Status**				
Single	4(3.6)	106(96.4)	23.319	<0.001^#^
Married/cohabiting	70(24.9)	211(75.1)		
**Religion**				
Christianity	21(8.7)	221(91.3)	43.467	0.001
Islam	53(35.6)	96(64.4)		
**Ethnicity**				
Yoruba	49(18.4)	218(81.6)	6.125	0.047^#^
Igbo	25(23.8)	80(76.2)		
Others	0(0.0)	19(100.0)		
**Qualification**				
RN	9(31.0)	20(69.0)	49.878	<0.001^#^
Post-basic	16(50.0)	16(50.0)		
Registered nurse/Midwife	39(22.3)	136(77.7)		
Registered midwife	10(21.7)	36(78.3)		
BNSc	0(0.0)	93(100.0)		
MSc and Postgraduate	0(0.0)	16(100.0)		
**Cadre**				
DDNS/ADNS/CNO	8(5.6)	135(94.4)	89.944	<0.001
PNO	47(53.4)	41(46.6)		
SNO/NOI/NOII	19(11.9)	141(88.1)		
**Years of Practice**				
1–5	36(37.9)	59(62.1)	65.538	<0.001^#^
6–10	19(31.7)	41(68.3)		
11–15	19(23.2)	63(76.8)		
16–20	0(0.0)	51(100.0)		
>20	0(0.0)	103(100.0)		
**Involved in treating severely malnourished child**				
Yes	42(15.6)	227(84.4)	6.165	0.013
No	32(26.2)	90(73.8)		

^#^Fisher’s exact p-value

Nutrition knowledge was also significantly associated with occupational characteristics of health care providers- cadre, duration of practice and involvement in treating severely malnourished child(ren). Respondents with BNSc and MSc/Postgraduate qualifications were significantly more knowledgeable than their counterparts without University degrees (p<0.001).

Table 4 shows the predictors of good nutrition knowledge. Respondents below 40 years of age have significantly less odds of good nutrition knowledge than those who were above 40 years. Single respondents were almost 9 times more likely to have good nutrition knowledge than the married respondents (CI = 8.791, 95% CI: 3.125–24.731). Using more than 10 years of work experience as a reference value, respondents with fewer years of experience had significantly less odds of good nutrition knowledge. Respondents in professional cadres DDNS/ADNS/CNO were about 2 times more likely to have good nutrition knowledge than the lower cadre. Christian respondents were almost 6 times more likely to have good nutrition knowledge than Muslims (OR = 5.810, 95% CI: 3.321–10.164).

**Table 4:** Predictors of good nutrition knowledge.

Variable	Odds ratio	95% CI		p-value
		Lower	Upper	
**Age (years)**				
21–30	0.310	0.109	0.882	<0.001
31–40	0.104	0.049	0.218	0.028
>40*	1			
**Marital status**				
Single	8.791	3.125	24.731	<0.001
Married*	1			
**Years of practice**				
1–5	0.143	0.077	0.268	<0.001
6–10	0.189	0.092	0.387	<0.001
>10*	1			
**Cadre**				
SNO/NOI/NOII*	1			
PNO	0.118	0.062	0.222	0.061
DDNS/ADNS/CNO	2.274	1.000	5.369	<0.001
**Ethnicity**				
Yoruba*	1			
Others	1.123	0.657	1.922	0.671
**Religion**				
Christianity	5.810	3.321	10.164	<0.001
Islam*	1			

*reference category

### Quality of nutrition education services

As shown in Table 5, it was observed that all 41(100.0%) of the centres had permanent seats in education venue, were organized for sessions, and had some form of teaching materials used in delivering nutrition education to pregnant women. Though they all had videos/TV screens, posters and charts for nutrition education, very few (22%), used visual aids during the sessions, had audio aids available (22%) or demonstrated food preparation with fresh foods (19.5%). In terms of message delivery, the health workers all stood in front of the mothers and gave information, and the pregnant women usually participated actively (85.4%). In 61% of the centres, the nurses/midwives met pregnant women on a one-on-one session when necessary. With regards to nutrition topics discussed, all the health workers talked about food and hand hygiene, importance of balanced diet and specific nutrients such as Folic Acid, Iron and Calcium. Sixty one percent (61%) discussed maternal nutrition in the presence of other health conditions such as hypertension and diabetes mellitus.

**Table 5:** Assessment of nutrition education facilities and services at the ante-natal clinics.

Variable	Yes (%)
**ANC services and clinic environment**	
Permanent seats in education venue	41(100.0)
Absence of noise and interference during the group session	8(19.5)
A conducive learning environment	41(100.0)
Midwife organized women to settle down for session	41(100.0)
Deliver education and counseling for diet and nutrition	41(100.0)
Recommend reading material about nutrition to pregnant women to read at home	33(80.5)
Encourage pregnant women to attend with spouses	41(100.0)
**Availability of guidelines and nutrition resources at the antenatal clinic**	
Have teaching materials used in delivering nutrition education to pregnant women	41(100.0)
Maternal nutrition guidelines for reference or guidance	25(61.0)
Use of visual aids in the session	9(22.0)
Availability of audio aids	9(22.0)
Videos/TV screen for ANC messages	41(100.0)
Availability of job aids, posters, and charts	41(100.0)
Fresh foods or demonstration of food preparation	8(19.5)
**Teaching strategies**	
During teaching the midwife stands in front of the mothers and give information with demonstrations of foods	41(100.0)
Midwives/ nurses meet pregnant women on a one-on-one session when necessary	25(61.0)
Active participation of mothers in nutrition teaching	35(85.4)
Adequate time for questions and answers for the pregnant women	25(61.0)
**Nutrition-related topics taught**	
Food hygiene (including hand hygiene)	41(100)
Increased intake of fruits and vegetables	41(100)
Balanced diet	41(100)
**Specific nutrients covered**	
Iron, Folic Acid, Calcium, Vitamins A, C, Carbohydrate, Protein, Fat	41(100)
Thiamin	16(39.0)
Ribovlavin	24(58.5)
Niacin	16(39.0)
Zinc	24(58.5)
Vitamin B12	32(78.0)
Speaks on nutrition requirement for clients with hypertension, diabetes, etc	25(61.0)
**Overall quality of prenatal nutrition education services**	
Adequate	25(61.0)
Inadequate	16(39.0)

## Discussion

Overall, majority of the respondents had good knowledge of nutrition in pregnancy and provided good nutrition education services, albeit some gaps. It can be inferred from the findings of the study that majority of nurses have been exposed to nutrition courses in the course of their training, so as to increase their knowledge on prenatal nutrition. It was rather surprising that less than half of them knew that Iron need increases during pregnancy, showing that the quality of nutrition education needs to improve in addition to re-fresher courses after graduation. In other similar studies in the African Region and beyond, respondents reported having exposure to maternal nutrition during their professional education [21, 23, 25]. On the contrary, midwives in Australia had a poor knowledge of nutrition knowledge. Though the completion rate (6.9%) of the online survey was very low, it showed that only about half of the midwives received nutrition education courses during their education [9].

The good knowledge of nutrition seen among respondents in this study could be attributed to their exposure to nutrition courses during nursing/midwifery program, further on-the-job nutrition related training, seminars and short courses. A high proportion had also been practicing for many years. Short-comings in some aspects of knowledge was however evident which may be due to studying some elective nutrition courses and the integration of nutrition-related topics into other courses. Similarly, in another study conducted in a Ghana hospital in Tamale Metropolis, Nurses’ knowledge of prenatal nutrition was average (54.0%) [26]. In another cross-sectional study on nutritional knowledge of Australian nurses in a hospital, the mean knowledge score for all nurses was good 60.2% [27]. In a postal survey, though with a very poor response rate, respondents had good knowledge of pre-natal nutrition and were confident on impacting nutrition knowledge on women [21].

Some other authors reported poor knowledge on nutritional needs during pregnancy among final year midwifery students in Ghana [22], and midwives in Sudan [25]. In developed countries like Australia and United Kingdom, midwives/nurses also showed poor knowledge of maternal nutrition [28, 29]. The cause of poor nutritional knowledge is linked to little emphasis given to nutrition education during the training of midwives [9, 30].

Multivariate analysis showed that good nutrition knowledge was predicted by older age, more work experience and higher cadre. Presumably, older respondents have more work experience and are more likely to be found in higher ranks among the cadre of professional nurses and midwives. This shows that knowledge acquisition goes beyond the nursing school and a lot is learnt on the job. The significantly better knowledge among respondents involved in management of severely malnourished children also supports this. Being single also predicts good nutritional knowledge signifying that the single respondents may have more time to study and participate in continuous professional development courses to improve their nutritional knowledge.

Based on the observation made during visits to the ANC centres, all the centres had permanent seats in education venue, conducive learning environment, midwives organized women to settle down for session, and they had teaching materials used in delivering nutrition education to pregnant women. The materials help to educate and expose pregnant women to importance of adequate care and nutrition during pregnancy. These observations are not surprising because provisions for smooth delivery of ANC services in the study locations by the State Government had been on-going. In another study carried out in a very busy maternity unit of a Teaching Hospital in Uganda [31], the clinic organization followed a similar pattern as there were permanent seats and portable benches. The maternal nutrition education sessions were embedded in the antenatal education which was reserved for pregnant women attending the ANC clinic for the first time in the current pregnancy. The pregnant women concerned were identified by the midwife and moved to the venue/education point and those who attended with their spouses were given a special corner for more comfort. In terms of resources and delivery, very little time was dedicated to nutrition in pregnancy. Also in that study, the midwives used direct verbal instruction method, there were no demonstrations and they hardly had teaching materials to enhance learning. They were also not aware of existing maternal nutrition guidelines and they lamented the insufficient training in nutrition for their expected role [31]. Our study showed that overall, about two-fifths of the facilities observed delivered inadequate prenatal nutrition education services. Some areas which require improvement with reference to offering prenatal nutrition education were revealed. Sessions should be less noisy, the nurses need to use more visual aids during the education sessions and also organize food demonstration sessions to improve on the clients’ nutrition knowledge and diet.

### Strengths and weaknesses of the study

This study reported the level of nutrition knowledge among nurses with the responsibility of rendering prenatal nutritional education services to a large number of women at the first level of care in Lagos. At the same time, it also assessed the quality of nutrition education and facilities available at the ANC clinics. There is dearth of data on these vital indices in Nigeria hence the importance of the study in filling this gap. It is hoped that the findings of this study will prompt the stakeholders in maternal and newborn health to intervene and bring the provision of nutrition education services and facilities at antenatal clinics to optimal levels.

There is the possibility of Hawthorne effect during clinic observations and this might have increased scores for the quality assessment. Lagos State is predominantly urban, and the study did not cover the minority rural areas where more gaps likely exist. The data could have been complemented with assessment of nutrition knowledge of the pregnant women at the ANC clinics. This could constitute future studies.

## Conclusion/Recommendations

Majority of the respondents had good knowledge of prenatal nutrition as many of them reported taking nutrition course in the nursing programme through elective classes and integration of nutrition-related topics into other courses. There was a statistically significant association between socio-demographic characteristics of respondents and level of nutrition education. Age, marital status, religion, cadre, years of practice and involvement in treatment of malnourished child were factors which significantly influenced nutritional knowledge of respondents.

Being married, Christian religion, DDNS/ADNS/CNO cadre predict good knowledge of nutrition. Overall, many requirements for good prenatal nutrition education services were met by the ANC clinics but there were also lapses in use of visual and audio aids and non-demonstration of food preparation.

It is necessary to have focused periodic refresher training sessions on prenatal nutrition and education for nurses and midwives with less experience, and those in lower cadre in order to bridge the gap with more senior colleagues. The involvement of Nutritionists in these trainings and also in client nutrition education in the ANC clinics could be explored. All necessary equipment required for health service providers to execute their roles as nutrition counselors and educators should also be provided by the government.

## Supporting information

S1 Data (XLSX)

S1 File (DOCX)
