# Supplementary material for: Pre-natal nutrition education: Health care providers’ knowledge and quality of services in primary health care centres in Lagos, Nigeria
Source: PLoS One. 2021 Nov 9;16(11):e0259237. doi: 10.1371/journal.pone.0259237 (PMC8577761; doi:10.1371/journal.pone.0259237)
Supplement: S1 File — (DOCX) [file pone.0259237.s002.docx]

**QUESTIONNAIRE**

**Pre-natal nutrition education: health care providers’ knowledge and quality of services in primary health care centres in Lagos, Nigeria**

Please tick (√) the option appropriately and give reasons for your answer where necessary

Do not write your name on the questionnaire.

**Serial No………….**

**Section A: Socio-demographic data**

1. Age in years (as at last birth day) ………………………………….
2. Marital Status (a) Single (b) Married/cohabiting (c) Divorced/Separated / Widow/widower
3. Religion (a) Christianity (b) Islam
4. Ethnicity? (a) Yoruba (b) Igbo (c) Hausa (d) Other… (please specify)

**Section B: Occupational Characteristics**

1. Qualifications (a) RN (b) post-basic (c) Registered Nurse / Midwife (d)Registered Midwife (e) BNSc (f) MSc and Postgraduate

(g) Others……….

1. Cadre (experience as nurse/midwife) (a) DDNS (b) ADNS (c) CNO (d) PNO (e) SNO (f) NOI (g) NOII
2. How many years altogether have you practiced your profession? ……………………
3. How long have you been working here? …………………
4. Are you involved in the treatment of severely malnourished child? (a) Yes (b) No

**SECTION C: Exposure to Nutrition Information**

**Nursing Programs**

1. In your nursing program, did you take a nutrition course? (a) Yes  (b) No 
2. In addition to a required nutrition course, were nutrition courses also offered as elective classes? (a) Yes  (b) No 
3. Were nutrition-related topics covered solely or integrated into other courses?

Solely  Integrated  Offered both 

1. Were pregnancy related diets covered in the nutrition-related curriculum? (a) Yes (b)No
2. Were modified diets covered in the nutrition-related curriculum? (a) Yes (b) No 
3. Was assisting pregnant women during mealtimes covered in the nutrition-related curriculum? (a) Yes  (b) No 
4. In clinical hours, do you recall the number of hours you teach nutrition related topics only?................
5. Do you feel your nursing program prepared you to answer pregnant women questions about nutrition and modified diets? (a) Yes  (b) No 
6. Do you recall the titles of any textbooks/manuals/handbooks used related to nutrition in your nursing program? (a) Yes  (b) No 
7. How many number of nutrition courses have you undertaken while in the midwifery school?..................
8. Have you received any refresher courses/training/seminar/ short courses on nutrition after midwifery school? (a) Yes  (b) No 
9. Are you aware of guidelines on nutrition? (a) Yes  (b) No 
10. Have you received training on guidelines on nutrition education for pregnant woman? (a) Yes (b) No 
11. Do you conduct nutrition assessment activities such as weight, height, etc for pregnant women? (a) Yes (b) No 

**SECTION D: Nutrition Education - Diet knowledge**

1. The regular/house diet should typically be composed of what type of food? Foods low in fat cholesterol and sodium  food high in fiber I do not know Others please specify
2. How many portions of milk and dairy products a day should be consumed in pregnancy? At least 2 portions  At least 3portions  At least 4 portions I do not know Others (please specify)
3. Inadequate and unbalanced nutrition during pregnancy affect who? Mother only Baby only Mother and Baby I don’t know Others please specify…
4. What happen to iron need of woman during pregnancy? Decreased Increased  Neither increased nor decreased I do not know Others (please specify)
5. Which seafood should be avoided during pregnancy? Fish Crab shrimp snailμtortoiseμI do not know Others (please specify)
6. During pregnancy period, there should be daily increase in the consumption of which type of food? Vegetables Fruits Fruit and VegetablesμI do not know Others (please specify)

1. During pregnancy there is need for increase in minerals such as …. Calcium Iron Zinc Iodine I do not know Others (please specify)
2. What causes softening of bones and deterioration of bone tissue (osteomalacia) during pregnancy? Inadequate consumption of minerals such as calcium and phosphorus in pregnancy  inadequate exposure to sunlightμI do not know Others (please specify)
3. What should mother do if she is overweight/obese in the pre-pregnancy period? Lose weight Reach normal range of BMI I do not know Others (please specify)
4. What causes neural tube defective births? Folate/Folic Acid inadequacy in pregnancy  Vitamin B inadequacy lack of multivitamin with 400 micrograms I do not know  Others (please specify)
5. What is the level of protein need of mother during pregnancy? Decrease Increase Neither decrease nor increase I do not know Others (please specify)
6. Which types of food is recommended for women with preeclampsia? Food rich in … SodiumZinc Magnesium  Iron Folates Low-fat milk Salt Sugar Don’t know Others…………..
7. Which types of food is recommended for client with diabetes mellitus? Protein  fruits vegetables carbohydrates fats Don’t know Others…………..

**APPENDIX 2**

**OBSERVATION CHECK LIST**

**ASSESSMENT OF QUALITY OF NUTRITION EDUCATION AT THE ANTENATAL CLINICS**

|  | **ASSESSMENTS OF QUALITY** | **Yes** | **No** |
| --- | --- | --- | --- |
| 1 | Permanent seats in education venue |  |  |
| 2 | Noise and interference during the group session |  |  |
| 3 | A conducive learning environment |  |  |
| 4 | Midwife organized women to settle down for session |  |  |
| 5 | Availability of guideline for delivery of diet and nutrition education |  |  |
| 6 | Deliver education and counseling for diet and nutrition |  |  |
| 7 | Have teaching materials used in delivering nutrition education to pregnant women |  |  |
| 8 | Recommend reading material about nutrition to pregnant women to read at home |  |  |
| 9 | Encourage Pregnant women to attend with spouses |  |  |
| 10 | Speak on nutrition requirement for clients with hypertension, diabetes, etc |  |  |
| 11 | **Availability of guidelines and nutrition education resources** |  |  |
| 12 | Use of visual aids in the session |  |  |
| a | Availability of audio aids |  |  |
| b | Videos/TV screen for ANC messages |  |  |
| c | Availability of job aids, posters, and charts |  |  |
| d | Fresh foods or demonstration of food preparation |  |  |
| e | Maternal nutrition guidelines for reference or guidance |  |  |
| f | **Teaching strategies** |  | |
| 13 | During teaching the midwife stands in front of the mothers and give information with demonstrations of foods |  | |
| a | Midwives/ nurses meet pregnant women on a one-on-one session when necessary |  |  |
| b | Minimal active participation by the mothers |  |  |
| c | There is limited time to ask question(s) by the pregnant women |  |  |
| d | **Taught Nutrient intake** |  |  |
| 14 | Protein |  |  |
|  | Fat |  |  |
|  | Iron |  |  |
|  | Calcium |  |  |
|  | Folic acid |  |  |
|  | Vitamin A |  |  |
|  | Vitamin C |  |  |
|  | Thiamin |  |  |
|  | Riboflavin |  |  |
|  | Niacin |  |  |
|  | Zinc |  |  |
|  | Vitamin B12 |  |  |
|  | **Food Hygiene** |  |  |
|  | Hand washing (when to wash hands) |  |  |
|  | Food / fruit and vegetable |  |  |
|  | Others |  |  |

Thank you for your participation.
